# Overview of Recent Trends in the Management of Metastatic Anal Cancer

**DOI:** 10.14740/wjon866w

**Published:** 2015-02-14

**Authors:** Mahender Yellu, Ayham Deeb, Olugbenga Olowokure

**Affiliations:** a Division of Hematology/Oncology, Stem Cell Transplantation, University of Cincinnati, Cincinnati, OH 45229, USA; b Division of Hematology/Oncology, University of Cincinnati, Cincinnati, OH 45229, USA

**Keywords:** Management, Metastatic anal cancer, Chemotherapy, Radiotherapy

## Abstract

Anal cancer is a relatively rare gastrointestinal tumor with roughly 7,000 new cases per year. Metastatic anal cancer as an initial presentation occurs in 10-20% of the patients. Treatment for localized disease is well established with concurrent chemoradiation (CCR) therapy as the standard of care; however, metastatic anal cancer remains a therapeutic challenge. National Comprehensive Cancer Network (NCCN) guidelines recommend systemic chemotherapy as the initial choice of treatment for metastatic anal disease. NCCN also recognizes the fact that there are limited data to influence the management of metastatic anal cancer but that some evidence suggests flouropyrimidine and cisplatin as the initial choice of treatment outside the setting of clinical trial. If the patient fails this regimen, options become limited with no strong level I evidence available to guide the treatment. We present two cases of metastatic anal cancer and discuss the potential treatment strategies after failing the initial systemic chemotherapy.

## Introduction

Management of metastatic anal cancer is challenging as treatment options are limited. Chemotherapy with cisplatin is generally used as the first-line agent but patients may progress or relapse even after achieving complete remission. Treatment of recurrent disease is difficult due to paucity of published data. Management of these patients is largely based on case reports and case series studies but large randomized studies are lacking. Recent reports of treating metastatic anal cancer patients with newer agents such as EGFR inhibitors were encouraging. We present two case reports of metastatic anal cancer and review the current knowledge of chemotherapeutic agents that are being used in this scenario.

## Case Reports

### Case 1

A 51-year-old white female with no significant past medical history presented to her primary care physician (PCP) with a 1-month history of worsening lower abdomen pressure, and low caliber stools. She had no rectal bleeding, change in appetite or weight loss. Prior to this, she was treated with steroid injections for pain in the coccyx area for 2 years. She had a history of bladder repair. Family history includes father with lymphoma and mother with history of stroke. Physical examination including digital rectal exam was unremarkable except for mild lower abdominal tenderness.

She underwent a transvaginal ultrasound and was noted to have a 4.3 cm mass arising from cervix. A follow-up computed tomography (CT) scan of the abdomen/pelvis showed exophytic mass possibly arising from rectum. Endoscopic ultrasound (EUS) revealed a mass pushing the rectum from outside and a colonoscopy showed an indurated mass in the lower rectum/anal area but no mucosal lesions. EUS-guided biopsy was positive for squamous cell cancer (SCC). Further analysis of specimen for KRAS mutation was negative. Given squamous histology and no mucosal erosions/lesions within the bowel, it was felt to be a cervical cancer initially; therefore she was referred to a gynecologist. Biopsies of the endometrium and cervix were negative for malignancy. A positron emission tomography (PET) scan confirmed the disease and additionally demonstrated extensive paraesophageal and supraclavicular lymph nodal involvement. She underwent biopsy of her supraclavicular lymph node and was consistent with SCC.

She was commenced on chemotherapy with cisplatin and 5-flourouracil (5-FU) and after receiving two cycles, chemoradiation therapy was initiated due to bulky and painful anal mass. Post-chemoradiation, chemotherapy with cisplatin and 5-FU was resumed and she was able to finish a total of seven cycles. A PET/CT scan post-chemotherapy demonstrated no active disease. However a surveillance PET/CT scan 2 months later showed recurrence of the cancer. She was restarted on cisplatin and 5-FU but she had further progression of the disease. She was enrolled in a phase II trial with oral rigosertib but continued to have progressive disease. She was then commenced on weekly carboplatin and paclitaxel and continued for about 3 months interrupted only by hospitalization for rectal bleeding. She then underwent salvage debulking surgery with diverting colostomy for intractable pain, fecal incontinence that was complicated by rectovaginal fistula. She had severe neoplastic pain, requiring high doses of narcotics and intrathecal pain pump. She was then started on salvage regimen with mitomycin and 5-FU and received two cycles but with progression of disease. She received further radiation therapy for the localized disease and now she is receiving cetuximab without irinotecan due to low platelet counts. Patient is currently alive at 26 months after the diagnosis of anal cancer.

### Case 2

An 84-year-old white healthy male presented to his PCP complaining of anal bleeding and swelling in the anus. On physical exam, the patient had palpable enlarged inguinal lymph node in the left groin in addition to palpable anal mass. He was referred to the rectal surgery clinic where anoscopy was performed. An anal mass was identified, biopsies were obtained and the pathology demonstrated SCC. The patient underwent staging workup with PET/CT scan which revealed bilateral inguinal, para-aortic and mediastinal lymphadenopathy in addition to local uptake. After a lengthy discussion, he was commenced on concurrent chemoradiation (CCR) therapy with 5-FU and mitomycin. He developed pancytopenia and severe anal skin burn from chemoradiation requiring prolonged break between the two chemotherapy cycles. He had a PET/CT scan 3 months after completion of chemoradiation therapy that demonstrated complete resolution of uptake in the primary mass in addition to decreased size and uptake in the inguinal and mediastinal lymphadenopathy. After a detailed discussion with the patient about the possibility of persistent disease, he opted for observation only.

## Discussion

### Anal cancer overview

Anal cancers are uncommon, comprise only 2.5% of gastrointestinal tumors and represent 0.4% of all new cancers [1]. According to cancer statistics, incidence of new anal cancers has been rising and is now approximately 1.8 per 100,000 (7,210) men and women per year whereas the death rate is 0.2 per 100,000 (950) men and women per year [1]. The five-year overall survival (OS) rates for localized, regional, unstaged and distant disease are approximately 80%, 58%, 60.4% and 32% respectively [1]. Anal cancers usually develop above the anal margin and those that occur below it are treated similar to that of squamous cell skin cancers. The predominant type of anal cancer is epitheloid or squamous type (80%), less commonly transitional and even less frequent is adenocarcinoma/melanoma [2].

The increased incidence of anal cancer is mainly attributed to high risk sexual behavior, increased transplantation rates, prolonged use of immunosuppressive agents and increased smoking [3-5]. Risk factors known to be associated with anal cancer include human papilloma virus (HPV) infection, immunosuppressive therapy, high risk sexual behavior, HIV infection and smoking [6]. Most common presentations of anal cancer include rectal bleeding, sensation of rectal mass, rectal mass, pain or pressure in the lower abdomen [7]. Apart from tissue biopsy which is considered the gold standard for diagnosis of anal cancer, imaging modalities such as CT scan, magnetic resonance imaging (MRI), PET scan and procedures including sigmoidoscopy, colonoscopy and endoscopic ultrasound remain useful.

### Management of non-metastatic anal cancer

Foundation for the revolutionary approach for the management of anal cancer was laid about four decades ago when Nigro et al used CCR to treat primary anal cancer [8]. This successful approach especially the opportunity to spare the anal sphincter is now considered the standard of practice whereas surgical resection (abdominoperineal resection) remains useful for salvage purposes. The significance of CCR was further confirmed in several non-randomized trials [9, 10]. Treatment response rate has exceeded over 80% with combined chemoradiotherapy according to European cooperative group study [11]. It was also found that the sex, skin ulceration and lymph node involvement could influence the OS.

CCR therapy with 5-FU and mitomycin is the initial choice of treatment in non-metastatic anal cancer [12, 13]. Additionally using two chemotherapy drug regimens such as mitomycin or cisplatin combined with 5-FU and radiotherapy (RT) has yielded better results than single drug regimen with RT or RT alone [5, 11, 12, 14-16]. CCR was also found to be superior to RT alone in multiple trials [11, 17]. Combination of 5-FU and cisplatin when compared with 5-FU and mitomycin was shown to have similar efficacy for locally advanced disease but the former was associated with more toxicity [18, 19]. In one study cisplatin-based chemotherapy showed no significant benefit over mitomycin in improving the disease free survival and was also associated with worse colostomy frees survival [20]. Induction chemotherapy prior to CCR has demonstrated no significant benefit [21, 22]. Radiation therapy alone achieved comparable survival rate to that of surgical intervention and even better results with increased dose but limited by potential toxicity [23].

With combined chemoradiation and salvage surgery, complete response (CR) can be achieved in over 90% of the patients [9, 11]. Though upfront surgical intervention has some advantages such as obtaining adequate margins and resection of nearby lymph nodes, significant morbidity and mortality associated with it makes it a less favorable initial choice [14]. With abdominoperineal resection the five-year OS can be > 50% in patients with persistent and recurrent anal cancer [24].

### Risk factors for relapse

Predicting tumor relapse can be difficult but sex and few tumor characteristics may assist in identifying the high risk group. Univariate and multivariate analysis identified female sex, HPV-16, tumor size, tumor histology, tumor stage at presentation, node positive disease, residual disease after treatment, and skin ulceration as the risk factors for recurrence [11, 12]. Compared to squamous cell anal cancer, adenocarcinoma of anal canal tends to be more aggressive and is associated with high relapse rate [25]. With surgical intervention, achieving negative margins is important but it may not protect against relapse [14]. Multiple trials demonstrated varying relapse rate but generally it ranges from 30% to 50% [15, 16]. Residual disease after CCR indicates increased risk of relapse [26]. Outcome could differ in residual disease compared to relapse with the later having worse survival [27].

### Management of metastatic presentation

Metastatic anal cancer at presentation is relatively less common and represents a therapeutic challenge. Local recurrence in anal cancer is about two times more common compared to distant metastasis [12, 25]. In one retrospective study of 92 patients with anal cancer, 17% of patients had local recurrence and 9% had distant metastases [12]. Distant metastasis in anal cancer tends to involve liver, lung, extra pelvic lymph nodes, peritoneum, and bones [13]. The cure rate is much greater when the disease has relapsed locally as opposed to distant metastasis [25]. In one study of 328 anal cancer patients, 73 patients had local recurrence, of whom 45% were treated with curative intent (surgical resection). The OS rates at 3 and 5 years were 79% and 66%, and cancer-specific survival rates at 3 and 5 years were 84% and 75% respectively [9].

Several questions including continued therapy until unacceptable toxicity is reached as opposed to terminating treatment after a defined number of chemotherapy cycles in metastatic anal cancer remain unanswered. Observation can be considered as was done in our second patient especially when there is discordance between clinical and images response. Cisplatin combined with 5-FU was used initially in three patients with metastatic anal cancer involving liver and achieved excellent response with marked tumor regression in all three patients [28]. Subsequently in another study with 19 metastatic anal cancer patients treated with cisplatin-based therapy and after a median of four cycles, 66% of the patients responded to the treatment with one CR and 11 partial response (PR). One- and five-year OS rates were 62.2% and 32.2% respectively [29]. NCCN guidelines recommend cisplatin as the initial regimen for distant metastatic anal cancer [30]. Treatment options are limited when patient fails this regimen and most clinicians base the therapy on their prior experience or published literature. Our first patient was enrolled in a clinical trial as a second line of therapy, but due to progressive disease she was initiated on carboplatin and paclitaxel. This combination regimen was used in case report prior achieving complete remission and the patient was disease free for 5 years [16]. Single agent paclitaxel was also used in one case series of seven patients (local, persistent and distant metastasis) and achieved sustained response for up to 6 months and OS up to 14 months since initiation of the therapy [31]. Similar results have been reported in another case series using paclitaxel [32]. However the dosing and frequency have differed in these two case series. Table 1 presents the OS in two large case series of metastatic anal cancer using cetuximab and irinotecan combination and single agent paclitaxel [31, 33].

**Table 1:** Chemotherapy Regimens for Metastatic Anal Cancer [31, 33]

Chemotherapy	Treatment	Prior treatment	Response	PFS (months)	OS (months)	KRAS status	Toxicity grade
Cetuximab/irinotecan	First	NA	NR	7	12	WT	2
Cetuximab/irinotecan	First	NA	NR	8	12	WT	1
Cetuximab/irinotecan	First	NA	PD	2	7	G12V mutation	0
Cetuximab/irinotecan	First	NA	PD	2	6	G12V & D33D mutation	0
Cetuximab/irinotecan	Second	5-FU, cisplatin	PR	10	21	WT	2
Cetuximab/irinotecan	Second	5-FU, cisplatin	PR	3.5	NA	WT	1
Cetuximab/irinotecan	Third	Capecitabine, mitomycin C, vinorelbine	PR	5	5	ND	1
Paclitaxel	First	NA	PD	2	7		
Paclitaxel	First	NA	PR	4	12		
Paclitaxel	First	NA	SD	4	6		
Paclitaxel	First	NA	CR	8	14		
Paclitaxel	First	NA	PR	6	NA		
Paclitaxel	First	NA	PD	2	5		
Paclitaxel	First	NA	PR	NA	NA		

5-FU: 5-fluorouracil; NA: not available; NR: no response; PD: progressive disease; PFS: progression free survival; PR: partial response; OS: overall survival; SD: stable disease.

Use of EGFR inhibitors has been reported in multiple patients recently with metastatic anal cancer. EGFR protein expression can be found in majority of the anal cancer patients but its mutation or amplification has not been identified [34]. In one study with 21 anal cancer patients, all patients demonstrated strongly positive EGFR overexpression (negative HER-2) but other markers such as p53, bcl-2 and cyclin D1 showed no specific pattern [35].

The preliminary results based on non-randomized case reports and cases series using cetuximab as a single agent or in combination with other agents in relapsed anal cancer are encouraging. However the benefit of cetuximab has not been consistent. In one case series of two patients, CR was achieved successfully in both the patients for 14 and 17 months with cetuximab and irinotecan [14]. Treatment with cetuximab and mitomycin demonstrated good response for 8 months in one case report [36]. Another case report achieved excellent results with no signs of disease progression 8 months after adding cetuximab to irinotecan when the disease was found to progress on single agent irinotecan [37].

In one case series of three patients, treatment with either cetuximab alone or when combined with mitomycin or irinotecan showed some response initially but the therapy was terminated either due to worsening symptoms or secondary to hypersensitivity reaction [38]. In this case series, one patient was switched to panitumumab due to severe hypersensitivity reaction. Cetuximab was found useful in only patients with wild type KRAS but not with those harboring mutations [33]. However the significance of testing all the patients with anal cancer for KRAS mutation has not been studied. Since our first patient continued to have progressive disease despite using multiple regimens, she was initiated on cetuximab.

### Conclusion

Here we present two cases of metastatic anal cancer with very different approaches to their management strategy based on the paucity of existing evidence in treating patients in this setting. As currently recommended by NCCN, a combination of cisplatin and 5-FU would apparently be a reasonable starting option but more clinical trials are certainly required to answer several questions related to metastatic anal cancer treatment especially if the disease progresses after the initial regimen. Randomized trials incorporating EGFR inhibitors might be a useful consideration especially in those with wild type KRAS tumors.
